# Changes in Corticospinal and Spinal Excitability to the Biceps Brachii with a Neutral vs. Pronated Handgrip Position Differ between Arm Cycling and Tonic Elbow Flexion

**DOI:** 10.3389/fnhum.2016.00543

**Published:** 2016-10-25

**Authors:** Davis A. Forman, Mark Richards, Garrick N. Forman, Michael W. R. Holmes, Kevin E. Power

**Affiliations:** ^1^School of Human Kinetics and Recreation, Memorial University of NewfoundlandSt. John’s, NL, Canada; ^2^Faculty of Health Sciences, University of Ontario Institute of TechnologyOshawa, ON, Canada; ^3^Faculty of Medicine, Memorial University of NewfoundlandSt. John’s, NL, Canada

**Keywords:** transmastoid, transcranial, MEP, CMEP, cycling, isometric, neutral, pronated

## Abstract

The purpose of this study was to examine the influence of neutral and pronated handgrip positions on corticospinal excitability to the biceps brachii during arm cycling. Corticospinal and spinal excitability were assessed using motor evoked potentials (MEPs) elicited via transcranial magnetic stimulation (TMS) and cervicomedullary-evoked potentials (CMEPs) elicited via transmastoid electrical stimulation (TMES), respectively. Participants were seated upright in front on arm cycle ergometer. Responses were recorded from the biceps brachii at two different crank positions (6 and 12 o’clock positions relative to a clock face) while arm cycling with neutral and pronated handgrip positions. Responses were also elicited during tonic elbow flexion to compare/contrast the results to a non-rhythmic motor output. MEP and CMEP amplitudes were significantly larger at the 6 o’clock position while arm cycling with a neutral handgrip position compared to pronated (45.6 and 29.9%, respectively). There were no differences in MEP and CMEP amplitudes at the 12 o’clock position for either handgrip position. For the tonic contractions, MEPs were significantly larger with a neutral vs. pronated handgrip position (32.6% greater) while there were no difference in CMEPs. Corticospinal excitability was higher with a neutral handgrip position for both arm cycling and tonic elbow flexion. While spinal excitability was also higher with a neutral handgrip position during arm cycling, no difference was observed during tonic elbow flexion. These findings suggest that not only is corticospinal excitability to the biceps brachii modulated at both the supraspinal and spinal level, but that it is influenced differently between rhythmic arm cycling and tonic elbow flexion.

## Introduction

Limb orientation and joint posture influence the planning and execution of motor programs, and the processes behind this neural modulation have been attributed to both cortical and spinal mechanisms. H-reflex amplitudes of the soleus are modulated by changes in posture (Gerilovsky et al., 1989; Hwang, 2002) as are H-reflexes and F-waves of the abductor digiti minimi (ADM; Ginanneschi et al., 2005). Changes in these measures are typically indicative of changes in spinal excitability, and in postural-related work, these are thought to be due to variations in afferent input (Barry et al., 2008). As joint positions are manipulated, all muscles that cross the joint experience changes in length, thereby modifying the afferent feedback they provide to the spinal cord. Changes in corticospinal excitability have also been demonstrated as a result of postural variations. The amplitudes of motor evoked potentials (MEPs) to the biceps brachii and posterior deltoid are influenced by changes in upper-limb position (Mogk et al., 2014). More recently, the amplitudes of MEPs and cervicomedullary evoked potentials (CMEPs) were examined in order to assess corticospinal and spinal excitability, respectively, to the biceps brachii during various upper-limb postures (Nuzzo et al., 2016). MEPs and CMEPs were elicited while the upper-limb was placed in static, resting positions that varied between supinated, neutral, and pronated forearm positions. Both MEPs and CMEPs were modulated as an effect of forearm position, suggesting enhanced spinal excitability was the driving mechanism. However, a commonality of the previously mentioned studies is that they have all been performed with the target muscle in either resting or tonic conditions. The influence of forearm posture on corticospinal and spinal excitability during rhythmic motor outputs is unknown.

Rhythmic motor outputs are produced, in part, by spinally-mediated central pattern generators (CPGs; Grillner, 1981; Jordan, 1998) which function to generate the phasic activation of functional antagonists in order to achieve smooth, alternating movement. A key component of this process is the reciprocal inhibition of antagonist spinal motoneurones during agonist activation. The influence of afferent input that would otherwise interrupt the rhythmicity of the output, such as unwanted activation of the stretch reflex, is mitigated (Tanaka, 1974; Crone et al., 1987; Nielsen et al., 1992; Pyndt et al., 2003). The reflexive mechanisms that have been proposed to account for the influence of posture on corticospinal and spinal excitability during resting and tonic conditions may therefore be differentially modulated during rhythmic motor outputs. However, this is not to imply that posture does not alter corticospinal excitability during to rhythmic tasks. Bressel et al. (2001) examined surface electromyography (EMG) of several muscles of the upper-limb while arm cycling with three different handgrip positions; supinated, pronated, and neutral. While the pattern of muscle activity was unchanged between positions, brachioradialis EMG amplitude was greatest in the neutral handgrip position, indicating an effect of posture on neuromuscular function. The neurophysiological mechanism(s) underlying this finding has yet to be examined.

The purpose of the present study was to examine corticospinal and spinal excitability to the biceps brachii while arm cycling with both neutral and pronated handgrip positions. In our first of two experiments, MEPs and CMEPs were elicited during the most- and least-active phases of the biceps brachii (i.e., elbow flexion and extension, respectively) while participants cycled on an arm-cycling ergometer using either neutral or pronated hand pedals. To determine whether our findings from *Experiment 1* were in fact do to the rhythmic nature of arm cycling, *Experiment 2* was conducted whereby MEPs and CMEPs were measured during tonic elbow flexion that was also performed with neutral and pronated handgrips. We hypothesized that: (1) corticospinal excitability, not spinal excitability, of the biceps brachii would be higher during arm cycling with a neutral vs. pronated handgrip position; and (2) both corticospinal and spinal excitability would be higher while performing tonic elbow flexion with a neutral handgrip position.

## Materials and Methods

### Ethical Approval

The procedures of the experiment were verbally explained to each volunteer prior to the start of the session. Upon addressing all inquiries, written consent was obtained. This research was conducted in accordance with the Helsinki Declaration. *Experiment 1* was approved by the Interdisciplinary Committee on Ethics in Human Research at Memorial University of Newfoundland (ICEHR#: 20150636) and *Experiment 2* was approved by the Research Ethics Board at the University of Ontario Institute of Technology (REB#: 15-042). Procedures were in accordance with the Tri-Council guideline in Canada and potential risks were fully disclosed to participants.

### Participants

Ten, right-handed, male volunteers (25.3 ± 5.2 years, 176.6 ± 3.7 cm, 86.1 ± 10.9 kg) and nine, right-handed, male volunteers (22.6 ± 2.4 years, 174.1 ± 4.5 cm, 84.8 ± 13.7 kg) partook in *Experiment 1* and *Experiment 2*, respectively. Participants had no known neurological impairments. Prior to both experiments, all volunteers completed a magnetic stimulation safety-checklist in order to screen for contraindications to magnetic stimulation (Rossi et al., 2009). Additionally, participants were required to complete a Physical Activity Readiness Questionnaire (PAR-Q+) in order to screen for any contraindications to exercise or physical activity.

### Experimental Set-Up

**Figure 1:**
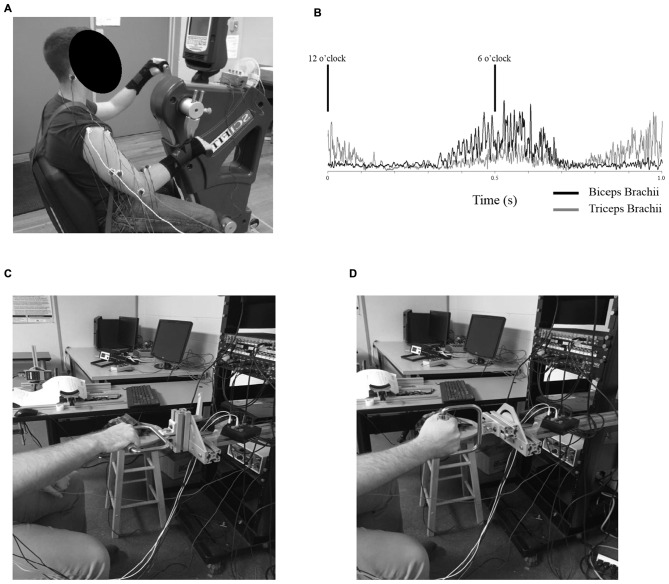
**(A)** Example of the experimental setup for *Experiment 1*. Participants were seated with their shoulders approximately in-line with the crank shaft of the ergometer while arm cycling at a constant cadence of 60 rpm and workload of 5% of their own peak power. Measurements were taken at the 6 o’clock (shown here) and the 12 o’clock position of the dominant arm with both neutral handgrip and pronated handgrip (shown here) positions. **(B)** Rectified electromyography (EMG) values for the biceps (black, solid trace) and triceps brachii (gray, solid trace) of a single participant throughout a single revolution of arm cycling. In this example, eight frames without stimulations were rectified and averaged over a 1 s window. Cycling cadence was set at a constant pace of 60 rpm (1 s representing one full revolution). The black lines denote the 6 and 12 o’clock positions. **(C,D)** Example of the experimental setup for *Experiment 2*. Participants were seated in front of a custom built apparatus and performed tonic, elbow flexions against a stationary handle. The handle could be adjusted to accommodate either a pronated handgrip (shown in **C**) or neutral handgrip (shown in **D**).

*Experiment 1* was conducted on an arm cycle ergometer (SCIFIT ergometer, model PRO2 Total Body, Tulsa, OK, USA) that could be fitted with either neutral or pronated hand pedals. Participants were seated upright at a comfortable distance from the hand pedals so that during cycling there was no reaching or variation in trunk posture. The seat height was adjusted so that the shoulders of each individual were level with the crank shaft of the ergometer. The hand pedals of the ergometer were fixed 180° out of phase for the entire duration of the protocol. All participants were required to wear wrist braces in order to limit the movement of the wrists during cycling as heteronymous reflex connections exist between the wrist flexors and biceps brachii (Manning and Bawa, 2011). Measurements were taken from two different pedal positions of the individual’s dominant arm; 6 and 12 o’clock relative to a clock face, whereby 12 o’clock was defined as the “top dead center” of the arm crank and 6 o’clock was defined as the “bottom dead center.” For example, 6 o’clock for a right handed participant would have been when their *right* hand was positioned at “bottom dead center” of the arm crank (see Figure 1A; right-handed participant at 6 o’clock). These two positions were chosen as they represent opposing activation phases of the biceps brachii during arm cycling (demonstrated in Figure 1B). Movement between 3 o’clock (when the elbow reaches full extension) and 9 o’clock (when the elbow reaches maximal flexion) occurs when the elbow is flexing and the biceps brachii is most active with peak EMG seen at approximately 6 o’clock. Movement between 9 o’clock and 3 o’clock occurs when the elbow is extending and the biceps brachii is minimally active with the lowest EMG seen at approximately 12 o’clock. Measurements at each position were taken separately. Participants were instructed to cycle at a constant cadence of 60 rpm and a constant workload of 5% of the individual’s own peak power with either a neutral or pronated handgrip position. The order of the two handgrip positions was randomized between participants (five participants started with neutral, five started with pronated). For both handgrip positions, measurements were taken at 6 and 12 o’clock, and the order of these two crank positions was also randomized.

*Experiment 2* was conducted on a custom-built apparatus (see Figures 1C,D) with a handle attachment that could be modified to either a horizontal or vertical orientation, corresponding to either a neutral or pronated handgrip position, respectively. The apparatus was secured to a wall. Participants were seated in front of the device with their dominant hand grasping the handle. The position of the dominant arm while holding the handle was simulated to correspond to the 6 o’clock position during arm cycling. In this position, participants were instructed to pull against the handle attachment in order to produce a pre-determined amount of biceps brachii EMG that corresponded to a similar amount of muscle activity the individual produced while arm cycling at the 6 o’clock position. Measurements were taken during this level of muscle activity for both neutral and pronated handgrips, the order of which was randomized.

### Electromyography

EMG activity of the biceps brachii and triceps brachii of the dominant arm were recorded using disposable bipolar Ag/AgCl surface electrodes (Meditrace 130, Kendall, Mansfield, MA, USA). Electrodes were positioned in line with the direction of the muscle fibers. For the biceps brachii, electrodes were placed on the midline of the muscle belly (over the junction between the long and short heads). For the triceps brachii, electrodes were placed on the lateral head. A ground electrode was placed on the lateral epicondyle and also on the dominant arm. Prior to electrode placement over recording sites, hair was removed via handheld razor, the skin was prepared by removal of dead epithelial cells (using abrasive paper) followed by sanitization with an isopropyl alcohol swab. EMG was sampled at 5 KHz using CED 1401 interface and Signal 5 (Cambridge Electronic Design Ltd., Cambridge, UK). Signals were amplified (gain of 300) and filtered using a 3-pole Butterworth with bandpass frequencies of 10–1000 Hz.

### Stimulation Conditions

Motor responses from the biceps brachii were elicited through three separate stimulation techniques: electrical stimulation at Erb’s point; transcranial magnetic stimulation (TMS); and transmastoid electrical stimulation (TMES). Stimulation intensities were determined while participants produced tonic elbow flexion of pre-determined intensity against a fixed handle for both experiments.

### Brachial Plexus Stimulation

The *M*_max_ of the biceps brachii was determined by eliciting M-waves through electrical stimulation of the brachial plexus at Erb’s point (DS7AH, Digitimer Ltd., Welwyn Garden City, Hertfordshire, UK). A pulse duration of 200 μs was used and intensities ranged from 100 to 250 mA (165.2 ± 49.5 mA). The cathode was placed in the supraclavicular fossa and the anode on the acromion process. The initial stimulation intensity was set at 25 mA and gradually increased until the elicited M-waves of the biceps brachii reached a plateau. The stimulation intensity that produced a plateau in M-wave amplitude was then increased by 10% to ensure maximal M-waves were elicited throughout the study. MEP and CMEP amplitudes (see below for stimulation paradigms) were normalized to the *M*_max_ elicited under the same experimental conditions in order to account for changes in peripheral neuromuscular excitability (Taylor, 2006).

### Transcranial Magnetic Stimulation

MEPs were elicited via TMS with a Magstim 200 (Magstim, Dyfed, UK). Stimulations were delivered over the vertex via a circular coil (13.5 cm outside diameter). Anatomically speaking, vertex is located on top of the cranium, mid-way between the right and left side of the participant’s skull and mid-way between their eyes and the back of their head. Vertex was determined by measuring the mid-point between the participant’s nasion and inion, and the mid-point between the participant’s tragi. The intersection of these two points was measured, marked and defined as vertex (Forman et al., 2014, 2015, 2016b; Pearcey et al., 2014; Copithorne et al., 2015; Philpott et al., 2015). The coil was held tangentially to the participant’s skull (approximately parallel to the floor) with the direction of the current flow preferentially activating the left motor cortex. The coil was held firmly against the participant’s head by one of the investigators to ensure careful and consistent alignment over vertex for each trial. Stimulation intensity was started at approximately 25% of maximum stimulator output (%MSO) and gradually increased until the average of eight MEP amplitudes equalled 15–20% of the individual’s own *M*_max_ (48.1 ± 10.1%MSO). This %MSO was used throughout the remainder of the experiment.

### Transmastoid Electrical Stimulation

TMES was delivered using Ag-AgCl surface electrodes applied just inferior to the mastoid processes. The pulse duration was fixed at 100 μs and stimulations intensities of 135–240 mA were used (189 ± 30.5 mA; DS7AH, Digitimer Ltd., Welwyn Garden City, Hertfordshire, UK). In order to ensure that TMS and TMES were activating similar portions of the corticospinal pathway, MEP and CMEP amplitudes were matched. TMES intensity began at 25 mA and gradually increased until the average of 8 CMEP amplitudes matched the average of the 8 MEP amplitudes previously elicited (~15–20% of the individual’s *M*_max_). This stimulation intensity was used throughout the remainder of the experiment.

### Experiment 1: Corticospinal and Spinal Excitability During Arm Cycling with Neutral and Pronated Handgrip Positions

Once surface electrodes were applied (see “Electromyography” Section), the maximal voluntary excitation (MVE) of the biceps and triceps brachii was determined during a maximal, 10 s, arm cycling sprint. Participants were seated behind a table-mounted Monark Wingate Testing ergometer (model 849E) that had been fitted with arm-cycling hand pedals. After a 2 min arm cycling warm-up at a self-selected cadence, participants were instructed to begin cycling as hard as they could. Upon reaching 100 rpm, a load equal to 5% of the individual’s bodyweight was automatically applied to the pedals. Participants continued cycling at a maximal effort for 10 s. Following this trial, peak power and the MVE of the biceps and triceps brachii were measured. MVE was taken from the root mean square (RMS) using a 25 ms moving average. Unlike isometric maximal voluntary contractions (MVCs), where muscle activity is relatively constant, maximal cycling results in alternating phases of high and low EMG, with a peak in EMG occurring once per revolution. The maximum value of these peaks from the RMS channel were summed and averaged from the middle 4 s of the 10 s trial (3–7 s; approximately 8–10 peaks, depending on the maximal cycling cadence of the individual). The middle 4 s were chosen to avoid the initial phase of the sprint involved in adjusting for the increased resistance and to avoid potential fatigue effects towards the end of the 10 s trial. Following a 10 min rest period, participants were moved to, and seated in front of, an arm cycling ergometer (Monark Rehab Trainer; model 881E) to determine stimulation intensities. With the hand pedal of the individual’s dominant arm locked at the 6 o’clock position and the hand pedal of the non-dominant arm locked at the 12 o’clock position, participants were instructed to produce 5% of their biceps brachii MVE by pulling their dominant arm against the locked pedal. Participants were shown a horizontal line on a computer monitor equal to 5% of their biceps brachii MVE (illustrated in an RMS channel) and were asked to reach and maintain that line. During this contraction, stimulation intensities for brachial plexus stimulation, TMS, and TMES were determined (see previous sections for methodologies).

Once stimulation intensities were determined, participants were moved to the SCIFIT ergometer. Participants were instructed to cycle at a constant cadence of 60 rpm against a constant workload equal to 5% of their own peak power, as determined during the 10 s sprint. Once steady-state had been achieved (maintained cadence of 60 rpm) a configuration consisting of eight MEPs and eight CMEPs was run at one of the two handgrip positions (neutral or pronated) and one of the two crank positions (6 or 12 o’clock). Stimulations were triggered automatically as the hand pedal reached the pre-determined crank position. The order of these stimulations was randomized throughout the configuration and stimulations were separated by approximately 7–8 s. To account for possible changes in the compound muscle action potential, a second configuration consisting of three Mwaves was performed immediately afterwards and were evoked under the same experimental parameters. These were also separated by 7–8 s. Completion of the two configurations (total of: 8 MEPS, 8 CMEPs, and 3 Mwaves) constituted the completion of one experimental condition. These steps were then repeated for the other hand crank position (6 or 12 o’clock) and handgrip position (neutral or pronated) for a total of four different experimental conditions (order randomized).

### Experiment 2: Corticospinal and Spinal Excitability During Tonic Contractions with Neutral and Pronated Handgrip Positions

*Experiment 2* was initiated following the completion of *Experiment 1*. In order to investigate whether the influence of handgrip position on corticospinal excitability during arm cycling was consistent across motor outputs, corticospinal excitability was assessed during tonic elbow flexion with neutral and pronated handgrip positions. It should be noted, however, that the comparison of the findings in *Experiment 1* to the findings in *Experiment 2* is not a direct comparison of rhythmic vs. tonic motor outputs. Rather it is an indirect comparison of the influence of handgrip position between tasks on corticospinal excitability. Due to equipment constraints, the EMG normalization procedures carried out in *Experiment 1*, as described above, could not be replicated for this follow-up study. However, we have recently shown that the biceps brachii EMG during arm cycling against a relative workload of 5% peak power is not significantly different than cycling against an absolute workload of 25W (Chaytor et al., 2014). Therefore, in order to match the intensity of the tonic contractions in *Experiment 2* as closely as possible with the intensity of the rhythmic motor outputs in *Experiment 1*, participants first performed a 30 s bout of arm cycling on a Monark Rehab Trainer (model 881E). Shoulder height was aligned with the height of the crank shaft and individuals were seated at a comfortable distance from the hand pedals so that there was no reaching or variation in trunk posture during cycling. Participants were instructed to cycle at a constant cadence of 60 rpm and against a constant workload of 25W for a continuous period of 30 s. The biceps brachii EMG was examined using an RMS with a 25 ms moving average. In this channel, the maximum values from each of the revolutions occurring in the middle 10 s of the 30 s trial (corresponding to approximately 10 revolutions) were measured and averaged. The peak RMS EMG of each revolution occurred during mid-elbow flexion, providing an approximate value that corresponded to the muscle activity seen at the 6 o’clock position of *Experiment 1*. Using a goniometer, the angles of the elbow and the shoulder were assessed for each participant while their dominant arm was statically placed at the 6 o’clock position of the arm cycling ergometer (shoulder angle: 40.6 ± 10.1° of shoulder flexion, elbow angle: 129.4 ± 10.7° of elbow extension). Participants were then moved to, and seated in front of, a custom built handle apparatus (Figures 1C,D). With the dominant hand grasping the handle, the elbow and shoulder angles that were previously measured on the arm cycle ergometer at the 6 o’clock position were replicated. These angles were held constant for the remainder of the experiment. The position of the chair was marked on the floor to ensure consistent positioning. Stimulation intensities were then determined during tonic elbow flexion. With the handle of the apparatus placed in the pronated orientation, participants were instructed to pull against the device to produce biceps brachii EMG equal to 50% of what they produced during their 30 s bout of arm cycling. This EMG magnitude was displayed as a horizontal line on a computer monitor that would also display the biceps brachii RMS in real time. Participants were instructed to match the target criteria by pulling against the handle to contract the biceps brachii. During this contraction, stimulation intensities for brachial plexus stimulation, TMS, and TMES were determined (see previous sections for methodologies).

Once stimulation intensities were determined, an EMG target corresponding to 100% of the EMG produced during arm cycling was placed on the screen. The handle apparatus was placed into either a neutral or a pronated orientation (order randomized between participants) and participants were instructed to pull against the handle in order to match and maintain that target. During this contraction, a configuration consisting of eight MEPs and eight CMEPs was performed in a randomized pattern. Stimulations were separated by approximately 7–8 s. As with *Experiment 1*, in order to account for possible changes in the compound muscle action potential, a second configuration consisting of three Mwaves was performed immediately after, in the same position and during the same level of contraction. Completion of the two configurations (total of: 8 MEPS, 8 CMEPs, and 3 Mwaves) consisted the completion of one experimental condition. These steps were repeated for the other handgrip position (neutral or pronated).

#### Data Analysis

Data was analyzed off-line using Signal 5 software (CED, UK). The peak-to-peak amplitudes of MEPs, CMEPs, and Mwaves of the biceps brachii were measured. The peak-to-peak amplitudes for all evoked potentials were measured from the initial deflection of the voltage trace from the background EMG to the return of the trace to background levels. Because changes in MEP and CMEP amplitudes can be the result of changes to the Mwave, both MEPs and CMEPs were normalized to the Mwaves evoked during the same experimental condition. Pre-stimulation EMG was measured from rectified traces and was defined as a 50 ms window of the mean rectified EMG immediately prior to the stimulation artifact (Forman et al., 2014, 2015, 2016b). Measurements were taken from the averaged files of all eight MEPs or all eight CMEPs.

#### Statistics

A two-way (handgrip position × crank position) repeated-measures ANOVA was used to assess whether statistically significant differences in MEP amplitudes, CMEP amplitudes (normalized to M-wave), or pre-stimulus EMG occurred as a main effect of handgrip position during arm cycling. Paired *t*-tests were then used to examine changes in amplitudes and pre-stimulus EMG between handgrip positions at the two hand crank positions. For *Experiment 2*, all data (MEP and CMEP amplitudes, as well as pre-stimulus EMG of the biceps and triceps brachii) was run as a *t*-test with repeated measures. All statistics were run on group data with a significance level of *P* < 0.05 and was performed using SPSS (V24, International Business Machines Corporation, Armonk, NY,USA). All data is reported as means ± SD and illustrated in figures as SE.

## Results

### Experiment 1: Corticospinal and Spinal Excitability During Arm Cycling with Neutral and Pronated Handgrip Positions

#### Corticospinal Excitability

##### MEPs

**Figure 2:**
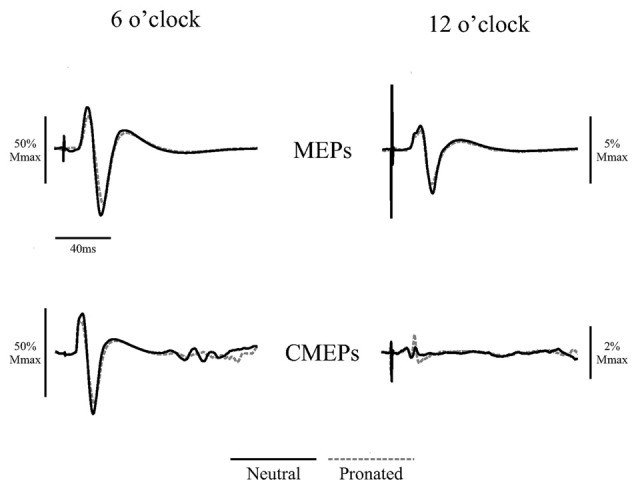
**Average traces of eight motor evoked potentials (MEPs) and eight cervicomedullary-evoked potentials (CMEPs) of the biceps brachii elicited during arm cycling at the two crank positions (6 and 12 o’clock) with a neutral handgrip (solid, black lines) and a pronated handgrip (dashed, gray lines) for a single, representative individual.** Average traces are normalized to the average of three Mwaves elicited during the same, experimental conditions.

**Figure 3:**
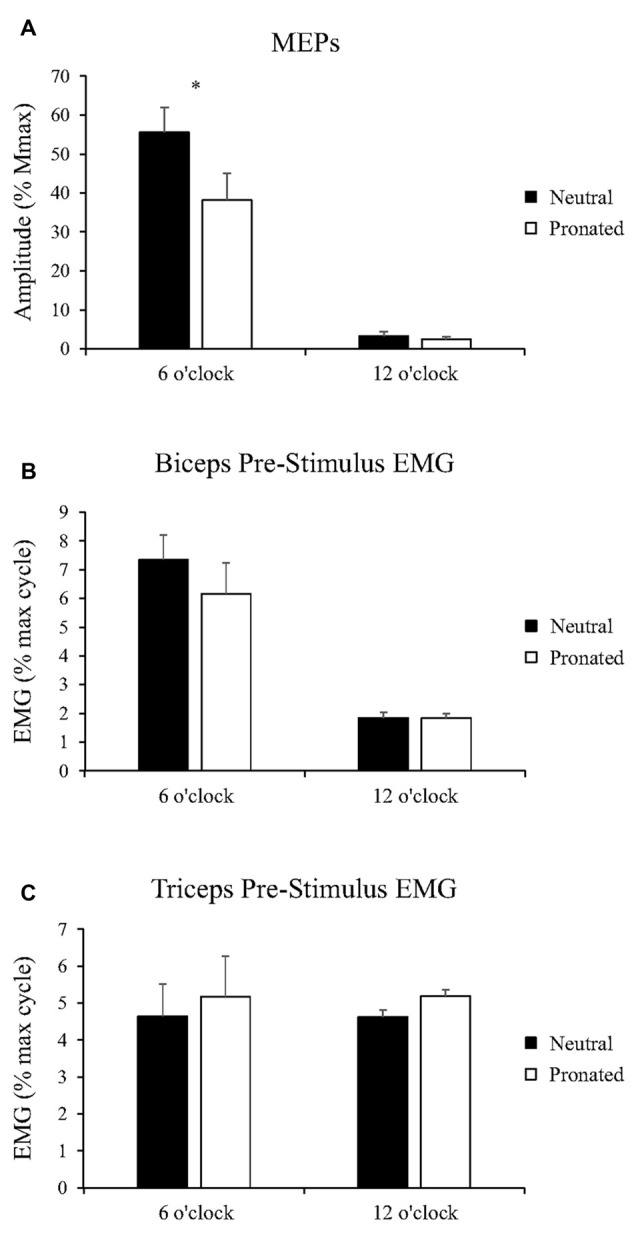
**Group data (mean ± SE, *n* = 10) during arm cycling for (A) MEP amplitudes of the biceps brachii, (B) biceps brachii pre-stimulus EMG prior to transcranial magnetic stimulation (TMS), and (C) triceps brachii pre-stimulus EMG prior to TMS.** Black bars correspond to measures taken during arm cycling with a neutral handgrip while white bars correspond to a pronated handgrip. MEP amplitudes are shown relative to the *M*_max_ taken during the same experimental condition. EMG is normalized to the maximum EMG found during the 10 s, maximal arm-cycling sprint. Asterisks denote a significant difference (*P* < 0.05) between the two handgrip positions.

Figure 2 (top panel) shows an example of the MEP amplitudes during arm cycling with neutral and pronated handgrip positions for both the 6 and 12 o’clock crank positions. In this example, MEPs (normalized to the *M*_max_) at the 6 o’clock crank position were 81.2 and 65.8% for the neutral and pronated handgrip positions, respectively. For the 12 o’clock crank position, MEPs were 5.3 and 3.6% for neutral and pronated handgrip positions, respectively. Group data (Figure 3A) demonstrated a main effect of handgrip position across crank positions, with MEP amplitudes larger while cycling with a neutral handgrip position. At the 6 o’clock crank position, MEP amplitudes were significantly larger while arm cycling with a neutral handgrip position compared with pronated (Neutral: 55.6 ± 20.2% of *M*_max_, Pronated: 38.2 ± 21.7% of *M*_max_, *P* < 0.05). While not statistically significant, the difference in MEP amplitudes at the 12 o’clock crank position between handgrip positions approached significance (Neutral: 3.3 ± 3.1% of *M*_max_, Pronated: 2.5 ± 2.1% of *M*_max_, *P* = 0.089).

##### EMG

Group data for pre-stimulus EMG of the biceps brachii and triceps brachii prior to MEPs (elicited via TMS) can be seen in Figures 3B,C, respectively. There was no main effect of handgrip position on pre-stimulus EMG for the biceps brachii. There was also no difference between handgrip positions at either 6 (Neutral: 7.3 ± 2.8% MVE, Pronated: 6.2 ± 3.4% MVE, *P* = 0.31) or 12 o’clock (Neutral: 1.8 ± 0.58% MVE, Pronated: 1.8 ± 0.55% MVE, *P* = 0.72). There was no main effect of handgrip position on pre-stimulus triceps brachii EMG. There was also no difference at the 6 (Neutral: 4.6 ± 1.6% MVE, Pronated: 5.2 ± 2.9% MVE, *P* = 0.54) or 12 o’clock position (Neutral: 4.6 ± 2.0% MVE, Pronated: 5.2 ± 2.9% MVE, *P* = 0.49).

#### Spinal Excitability

##### CMEPs

**Figure 4:**
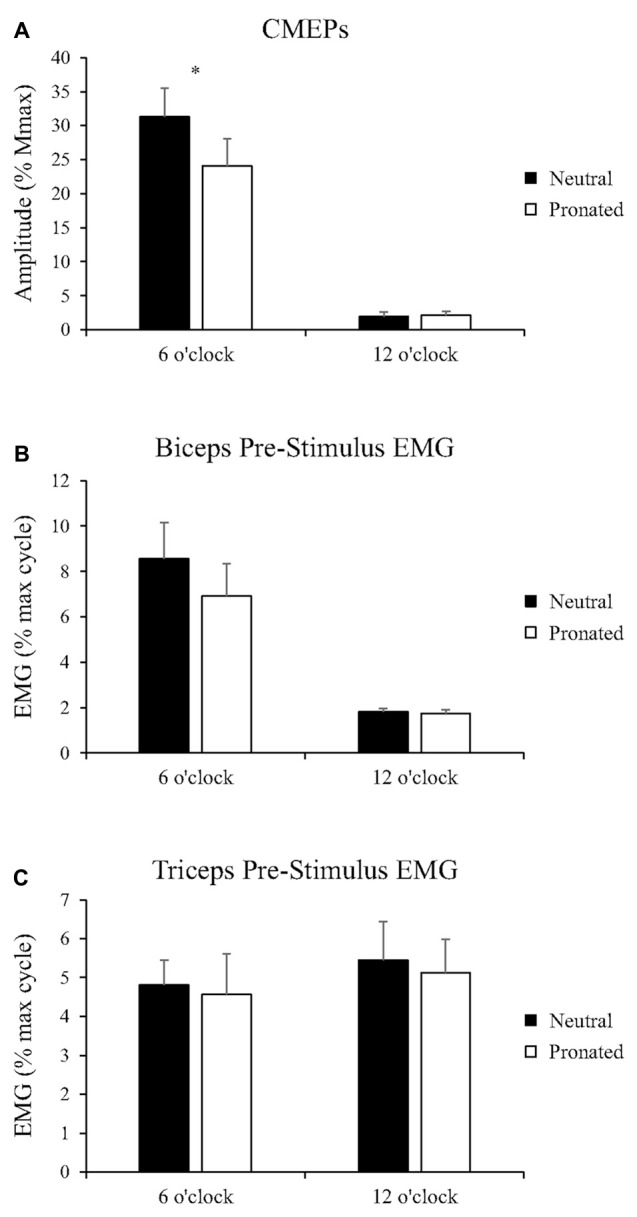
**Group data (mean ± SE, *n* = 8) during arm cycling for (A) CMEP amplitudes of the biceps brachii, (B) biceps brachii pre-stimulus EMG prior to transmastoid electrical stimulation (TMES), and (C) triceps brachii pre-stimulus EMG prior to TMS.** Black bars correspond to measures taken during arm cycling with a neutral handgrip while white bars correspond to a pronated handgrip. MEP amplitudes are shown relative to the *M*_max_ taken during the same experimental condition. EMG is normalized to the maximum EMG found during the 10 s, maximal arm-cycling sprint. Asterisks denote a significant difference (*P* < 0.05) between the two handgrip positions.

Figure 2 (bottom panel) shows an example of the CMEP amplitudes between arm cycling with a neutral vs. pronated handgrip position for both the 6 and 12 o’clock crank positions. In this example, CMEPs (normalized to the *M*_max_) at the 6 o’clock crank position were 56.5 and 46.3% for the neutral and pronated handgrip positions, respectively. For the 12 o’clock crank position, CMEPs were 0.41 and 1.1% for neutral and pronated handgrip positions, respectively. Group data (Figure 4A) demonstrated a main effect of handgrip position across crank positions, with CMEP amplitudes larger while cycling with a neutral handgrip position. At the 6 o’clock crank position, CMEP amplitudes were significantly larger while arm cycling with a neutral handgrip position compared with pronated (Neutral: 31.3 ± 11.9% of *M*_max_, Pronated: 24.1 ± 11.4% of *M*_max_, *P* < 0.05). CMEP amplitudes at the 12 o’clock crank position between handgrip positions were not significantly different (Neutral: 1.9 ± 1.5% of *M*_max_, Pronated: 2.1 ± 1.3% of *M*_max_, *P* = 0.54).

##### EMG

Group data for pre-stimulus EMG of the biceps brachii and triceps brachii prior to CMEPs (elicited via TMES) can be seen in Figures 4B,C, respectively. There was no main effect of handgrip position on pre-stimulus EMG for the biceps brachii. Pre-stimulus EMG was also not different between handgrip positions for either 6 (Neutral: 8.6 ± 4.5% MVE, Pronated: 6.9 ± 4.0% MVE, *P* = 0.32) or 12 o’clock (Neutral: 1.8 ± 0.43% MVE, Pronated: 1.7 ± 0.44% MVE, *P* = 0.21). There was no main effect of handgrip position on pre-stimulus EMG for the triceps brachii. There was also no difference for either 6 (Neutral: 4.8 ± 1.8% MVE, Pronated: 4.6 ± 3.0% MVE, *P* = 0.73) or 12 o’clock (Neutral: 5.5 ± 2.8% MVE, Pronated: 5.1 ± 2.5% MVE, *P* = 0.71).

### Experiment 2: Corticospinal and Spinal Excitability During Tonic Contractions with Neutral and Pronated Handgrip Position

#### Corticospinal Excitability

##### MEPs

**Figure 5:**
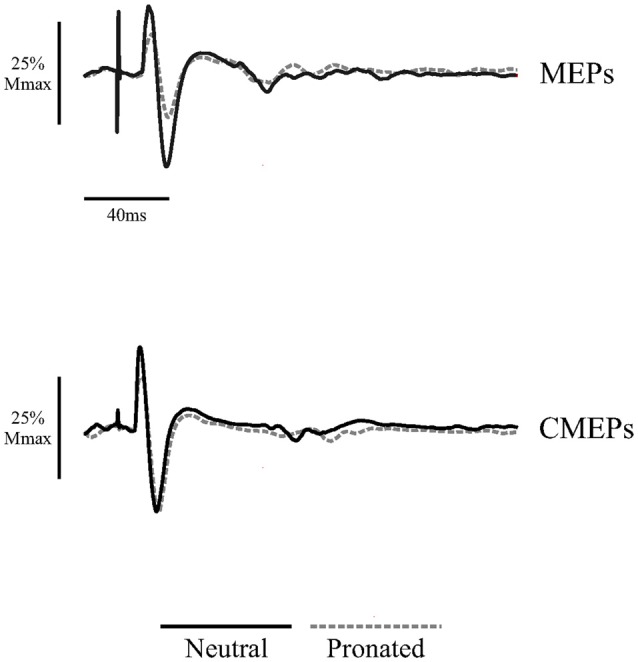
**Average traces of eight MEPs and eight CMEPs of the biceps brachii elicited during tonic elbow flexion with a neutral handgrip (solid, black lines) and a pronated handgrip (dashed, gray lines) for a single, representative individual.** Average traces are normalized to the average of three Mwaves elicited during the same, experimental conditions.

**Figure 6:**
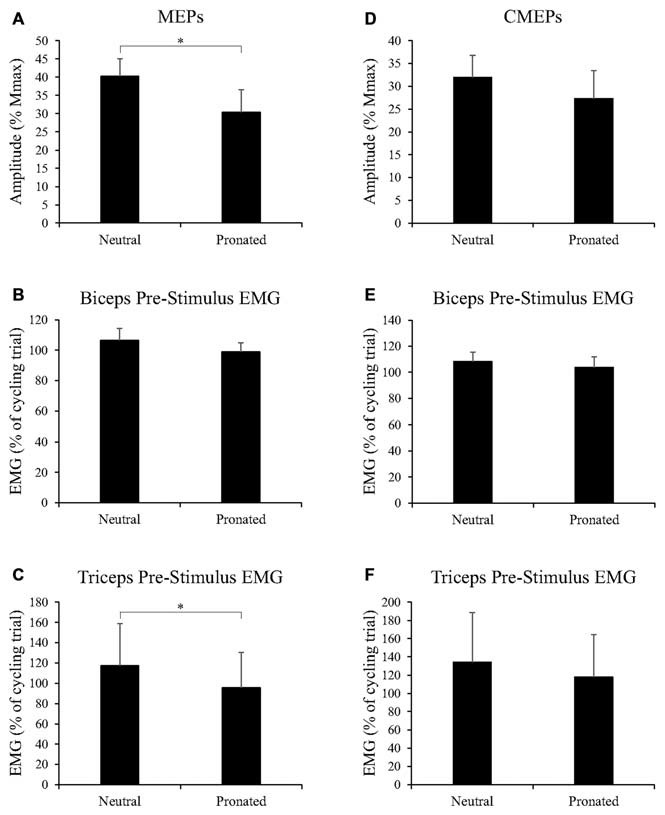
**Group data (mean ± SE, *n* = 9) during tonic elbow flexion for (A) MEP amplitudes of the biceps brachii, (B) biceps brachii pre-stimulus EMG prior to TMS, and (C) triceps brachii pre-stimulus EMG prior to TMS, as well as group data (mean ± SE, *n* = 7) during tonic elbow flexion for (D) CMEP amplitudes of the biceps brachii, (E) biceps brachii pre-stimulus EMG prior to TMES, and (F) triceps brachii pre-stimulus EMG prior to TMES.** MEP and CMEP amplitudes of the biceps brachii are expressed relative to the *M*_max_ taken during the same experimental conditions while EMG is normalized to the average EMG found during the 30 s, arm-cycling trial against a constant load of 25W and a constant cadence of 60 rpm. Asterisks denote a significant difference (*P* < 0.05) between handgrip positions.

Figure 5 (top panel) shows an example of MEP amplitudes during tonic elbow flexion with a neutral vs. pronated handgrip position while the participant pulled on the handle apparatus. In this example, MEPs (normalized to the *M*_max_) were 39.3 and 20.6% for the neutral and pronated handgrip positions, respectively. Group data (Figure 6A) demonstrated that the MEP amplitudes were significantly larger during a tonic contraction with a neutral handgrip position compared with pronated (Neutral: 40.2 ± 14.5% of *M*_max_, Pronated: 30.4 ± 11.2% of *M*_max_, *P* < 0.05).

##### EMG

Group data for pre-stimulus EMG of the biceps brachii and triceps brachii prior to MEPs (elicited via TMS) can be found in Figures 6B,C, respectively. Pre-stimulus EMG for the biceps brachii was not different between handgrip positions (Neutral: 106.5 ± 23.2% of cycling trial, Pronated: 98.9 ± 18.2% of cycling trial, *P* = 0.22). However, for the triceps brachii, pre-stimulus EMG was greater with a neutral handgrip position (Neutral: 117.4 ± 124.3% of cycling trial, Pronated: 95.8 ± 104.3% of cycling trial, *P* < 0.05).

#### Spinal Excitability

##### CMEPs

Figure 5 (bottom panel) shows an example of CMEP amplitudes during tonic elbow flexion with a neutral vs. pronated handgrip position while the participant pulled on the handle apparatus. In this example, CMEPs (normalized to the *M*_max_) were 40.3 and 32.7% for the neutral and pronated handgrip positions, respectively. Group data (Figure 6D) demonstrated that the CMEP amplitudes were not significantly different during a tonic contraction with a neutral handgrip position compared with pronated (Neutral: 32.0 ± 16.3% of *M*_max_, Pronated: 27.3 ± 9.3% of *M*_max_, *P* = 0.38).

##### EMG

Group data for pre-stimulus EMG of the biceps brachii and triceps brachii prior to CMEPs (elicited via TMES) can be found in Figures 6E,F, respectively. There were no significant differences for pre-stimulus EMG between handgrip positions for the biceps (Neutral: 108.4 ± 18.3% of cycling trial, Pronated: 103.8 ± 21.3% of cycling trial, *P* = 0.42) or triceps brachii (Neutral: 134.3 ± 143.8% of cycling trial, Pronated: 117.9 ± 123.2% of cycling trial, *P* = 0.24).

## Discussion

This study is the first to demonstrate that corticospinal excitability to the biceps brachii is higher while arm cycling with a neutral rather than pronated handgrip position. The findings of *Experiment 1* suggest that higher corticospinal excitability during cycling using a neutral handgrip may be driven by spinal mechanisms. In contrast, the findings of *Experiment 2* demonstrate that although corticospinal excitability is also higher with a neutral handgrip during *tonic* contraction, the increase may be primarily due to supraspinal mechanisms. Thus, handgrip positions have differential influences on corticospinal excitability between rhythmic arm cycling and tonic elbow flexion.

### Handgrip Dependent Changes in Corticospinal Excitability

In the present study, corticospinal excitability was significantly higher with a neutral rather than pronated handgrip for both arm cycling and tonic elbow flexion. These findings are supported by previous literature that has examined the influence of forearm posture on corticospinal excitability. Recently, Nuzzo et al. (2016) assessed corticospinal and spinal excitability to the biceps brachii while forearm orientation was manipulated. MEP amplitudes were larger with a neutral rather than pronated forearm position. CMEP amplitudes were not different between neutral and pronated positions, however, there was an overall effect of forearm position with CMEP amplitudes increasing in size from pronation to supination (smallest during pronation, largest during supination). The authors concluded that the observed changes in corticospinal excitability were likely spinal in origin (Nuzzo et al., 2016). This may also be the case in our study as MEPs and CMEPs were similarly modulated with both measures higher during arm cycling with a neutral handgrip position. However, the results of *Experiment 2*, which demonstrated an increase in corticospinal excitability in the absence of an increase in spinal excitability, indicates that supraspinal mechanisms are also influenced by handgrip position. This finding is supported by Perez and Rothwell (2015) who recently showed that MEPs of the first dorsal interosseous (FDI) increased during a simple gripping task when the forearm was in a neutral position compared to both supination and pronation. CMEP and F-wave amplitudes were unchanged, indicating that supraspinal factors were the driving mechanism behind the findings. Similar results have been found in hand and forearm muscles whereby manipulation of shoulder positon results in changes to TMS-induced stimulus response curves (SRCs; Ginanneschi et al., 2006; Mogk et al., 2014; Forman et al., 2016a). Intracortical facilitation (ICF) is a contributing mechanism behind these findings as increased ICF occurs with an increase in the slope of SRCs (Dominici et al., 2005; Ginanneschi et al., 2005). Increased ICF during a neutral handgrip position may explain the increase in supraspinal excitability in the present study for tonic elbow flexion, and possibly the increase in corticospinal excitability during arm cycling.

It is also worth noting in the present study that MEPs were larger for both experiments in the neutral handgrip position despite no difference in pre-stimulus biceps brachii EMG. As EMG represents the overall output of the motoneurone pool, this begs the question as to how corticospinal excitability was modulated while there was no apparent change in the muscle activity of the biceps brachii between handgrip positions. This could be due to the activation patterns of other synergistic muscles (Bressel et al., 2001). Bressel et al. (2001) demonstrated that the EMG of the brachioradialis, but not the biceps brachii, was significantly increased while arm cycling with a neutral compared to pronated handgrip position. Mechanical assessments have shown that the brachioradialis and biceps brachii share remarkably similar functions (Ettema et al., 1998). Both the brachioradialis and the biceps brachii exhibit significant force decrements during maximal elbow flexion tasks in the pronated forearm position (Jørgensen and Bankov, 1971), while producing the greatest amount of muscle activity during combined elbow flexion-forearm supination tasks (Cnockaert et al., 1975). The optimal moment arm for the brachioradialis may not be an anatomically neutral forearm position, but rather 30° of forearm supination (Ettema et al., 1998). Considering the mechanical similarities, and close spatial proximity within the primary motor cortex (Plow et al., 2010), it is possible that the higher activation levels of the brachioradialis during arm cycling with a neutral handgrip position (Bressel et al., 2001) resulted in increased excitability to the cortical neurones representing the biceps brachii. This would seemingly add to the growing body of literature that suggests the central nervous system initiates motor programs though the activation of muscular synergies (Park et al., 2001; Graziano et al., 2002; Plow et al., 2010) in order to account for the degrees of freedom problem (Bernstein, 1967).

### Handgrip Dependent Changes in Spinal Excitability

In the present study, spinal excitability was higher during arm cycling with a neutral compared to pronated handgrip position, however, spinal excitability was not influenced by handgrip position during tonic elbow flexion. For arm cycling, a possible mechanism for the lower spinal excitability using the pronated handgrip is heteronymous inhibition. Sub-threshold, electrical stimulation of the radial nerve inhibits the biceps brachii by delaying the discharge time of individual motor units (Naito, 2004). This is likely due to activation of group one afferents from the brachioradialis that project oligosynaptic inhibition to motoneurones of the biceps brachii. Heteronymous inhibition from the brachioradialis has a graded response to forearm position with interspike intervals in the biceps brachii shortest during forearm supination and longest during forearm pronation (Barry et al., 2008). Nuzzo et al. (2016) suggested that this mechanism was responsible for the results of their study which found a main effect of forearm position on CMEP amplitudes to the biceps brachii. In the present study, this mechanism could explain the lower spinal excitability observed while arm cycling with a pronated vs. a neutral handgrip. However, this inhibitory pathway has been characterized during tonic motor outputs, which may not persist during arm cycling. Indeed, activity of group one afferents has been shown to be mitigated via reciprocal inhibition during cycling (Tanaka, 1974; Crone et al., 1987; Nielsen et al., 1992). It should also be noted that this mechanism is unlikely to be present during arm cycling but not tonic elbow flexion whereby no decrease in spinal excitability at the pronated position was observed.

An additional consideration may be the activation of the 1a afferent pathway to the biceps brachii. As the biceps brachii is the principal supinator of the forearm (Cnockaert et al., 1975; Kulshreshtha et al., 2007) its muscle length is therefore longest when the forearm is pronated, and 1a afferents are most active in lengthened muscles (Burke et al., 1978). However, in the present study, CMEP amplitudes were smallest when the biceps brachii was at its longest muscle length (pronated handgrip) for arm cycling, whereas there was no change in CMEP amplitude between handgrip positions for tonic elbow flexion. This finding is consistent with previous studies where biceps brachii CMEP amplitudes were largest at short muscle lengths during resting conditions (Nuzzo et al., 2016). Similar results have been demonstrated in H-reflexes of the soleus (Gerilovsky et al., 1989; Hwang, 2002). This is not to say that 1a afferents were inconsequential in the present, experimental design. The rate of discharge of 1a afferents increases with external loading for both shortening and lengthening contractions (Burke et al., 1978), but the pattern of discharge becomes progressively less dependent on the length of the muscle. It is thus possible that the contraction intensity in the current study was sufficient enough to nullify the influence of muscle length on 1a afferent activity that is commonly seen during passive stretching.

The change in spinal excitability across handgrip positions during arm cycling, and lack thereof during a tonic contraction, may have been a result of the bilateral nature of arm cycling. Bilateral arm cycling increases the EMG of the contralateral limb (Vasudevan and Zehr, 2011). In the present study, the activity of the bilateral limb during arm cycling may have increased the excitability of the motoneurone pool to the biceps brachii in the contralateral limb. In the presence of other spinal pathways, such as heteronymous inhibition, which favor a neutral forearm orientation (Barry et al., 2008), this increased excitability may have contributed to the larger CMEP amplitudes in the neutral handgrip position. The tonic contractions, absent of a bilateral component, may have lacked the necessary excitation to demonstrate an effect of handgrip position on spinal excitability. Further investigation is required to explore this possible mechanism.

### Methodological Considerations

There are additional factors that should be considered when interpreting the differences in handgrip-influenced spinal excitability between rhythmic arm cycling and tonic elbow flexion. While all possible attempts were made in order to ensure that the level of muscle activation in *Experiment 1* (arm cycling) was replicated in *Experiment 2* (tonic elbow flexion), similar muscle activation does not necessarily correspond with similar levels of muscle loading across contraction types (Linnamo et al., 2003). In the present study, where EMG was similar between experiments, it is possible that there was greater muscle loading on the biceps brachii during tonic elbow flexion than rhythmic arm cycling. As external loading increases 1a afferent discharge and dampens the feedback of muscle length (Burke et al., 1978), the influence of handgrip position on spinal excitability may have been reduced. A second consideration is the possibility of muscle activity differences between the short and long heads of the biceps brachii. Motor unit recruitment thresholds and motor unit discharge rates are both influenced by forearm posture, but this influence is specific to the individual heads of the biceps brachii (Harwood et al., 2010). Whether this mechanism is altered between motor tasks (rhythmic vs. tonic contractions) and manifested in the results of the present study remains be to examined.

## Conclusion

Increased corticospinal and spinal excitability occurred while arm cycling with a neutral handgrip position, whereas only corticospinal excitability increased during tonic elbow flexion with the same handgrip position. This is the first instance where a rhythmic motor output has been reported to be influenced by different handgrips. In *Experiment 1*, corticospinal excitability was enhanced in part by a similar increase in spinal excitability, but quite likely by supraspinal factors as well. This may be the result of enhanced activation of synergistic elbow flexors acting upon the cortical neurons to the biceps brachii. The most plausible explanation for the increase in spinal excitability during arm cycling with a neutral handgrip is decreased heteronymous inhibition from the brachioradialis. This candidate warrants further investigation.

## Author Contributions

All authors participated in data collection, analysis and manuscript preparation. All authors have approved the submitted version of this manuscript.

## Funding

This work was supported by a grant to KEP from the Natural Sciences and Engineering Research Council of Canada (NSERC -#RGPIN-2016-03646). MWRH was also supported by a NSERC grant (NSERC - #RGPIN-2015-05765). DAF was supported by a NSERC Canada graduate scholarship (CGS-D).

## Conflict of Interest Statement

The authors declare that the research was conducted in the absence of any commercial or financial relationships that could be construed as a potential conflict of interest.
